# Human resources for health in Botswana: The results of in-country database and reports analysis

**DOI:** 10.4102/phcfm.v6i1.716

**Published:** 2014-11-21

**Authors:** Oathokwa Nkomazana, Wim Peersman, Merlin Willcox, Robert Mash, Nthabiseng Phaladze

**Affiliations:** 1 Faculty of Medicine, Department of Surgery, University of Botswana, Botswana; 2 Faculty of Medicine and Health Sciences, Department of Family Medicine and Primary Health Care, Ghent University, Belgium; 3 Division of Medical Sciences, Department of Primary Care Health Sciences, University of Oxford, United Kingdom; 4 Division of Family Medicine and Primary Care, Faculty of Medicine and Health Sciences, Stellenbosch University, South Africa; 5 School of Nursing, Faculty of Health Sciences, University of Botswana, Botswana

## Abstract

**Background:**

Botswana is a large middle-income country in Southern Africa with a population of just over two million. Shortage of human resources for health is blamed for the inability to provide high quality accessible health services. There is however a lack of integrated, comprehensive and readily-accessible data on the health workforce.

**Aim:**

The aim of this study was to analyse the existing databases on health workforce in Botswana in order to quantify the human resources for health.

**Method:**

The Department of Policy, Planning, Monitoring and Evaluation at the Ministry of Health, Ministry of Education and Skills Development, the Botswana Health Professions Council, the Nursing and Midwifery Council of Botswana and the in-country World Health Organization office provided raw data on human resources for health in Botswana.

**Results:**

The densities of doctors and nurses per 10 000 population were four and 42, respectively; three and 26 for rural districts; and nine and 77 for urban districts. The average vacancy rate in 2007 and 2008 was 5% and 13% in primary and hospital care, respectively, but this is projected to increase to 53% and 43%, respectively, in 2016. Only 21% of the doctors registered with the Botswana Health Professions Council were from Botswana, the rest being mainly from other African countries. Botswana trained 77% of its health workforce locally.

**Conclusion:**

Although the density of health workers is relatively high compared to the region, they are concentrated in urban areas, insufficient to meet the projected requirements and reliant on migrant professionals.

## Abstrait

**Ressources humaines pour les services de santé au Botswana: les résultats des bases de données et de l'analyse des rapports du pays**.

**Contexte:**

Le Botswana est un vaste pays à revenus intermédiaires d'Afrique australe avec un peu plus de 2 millions d'habitants. La pénurie de ressources humaines dans le domaine de la santé est rendue responsable de l'incapacité à fournir des services de santé accessibles et de haute qualité. Cependant, on manque de données accessibles, complètes et intégrées sur les professionnels de la santé.

**Objectif:**

L'objectif de cette étude était d'analyser les bases de données existantes sur les professionnels de la santé au Botswana afin de quantifier les ressources humaines de santé.

**Méthode:**

Le Département de Politique, Planification, Contrôle et Evaluation au Ministère de la Santé, le Ministère de l'Education et du Développement des Compétences, l'Association des Professionnels de la Santé du Botswana, L'Association des Infirmières et Sages-femmes du Botswana et le bureau de l'Organisation mondiale de la Santé dans le pays ont fourni des données brutes sur les Ressources humaines de Santé au Botswana.

**Résultats:**

La densité de docteurs et infirmières par 10 000 habitants était de 4 et 42, respectivement, 3 et 26 dans les zones rurales et 9 et 77 dans les zones urbaines. Le taux moyen de vacance de postes en 2007/2008 était respectivement de 5% et 13% dans les soins primaires et les soins hospitaliers, mais on s'attend à ce qu'ils atteignent respectivement 53% et 43% en 2016. Seuls 21% des docteurs inscrits à l'Association des professionnels de la santé étaient du Botswana, le reste venait surtout d'autres pays africains. Le Botswana a formé localement 77% de ses professionnels de santé.

**Conclusion:**

Bien que la densité des professionnels de santé soit relativement élevée par rapport à la région, ils sont concentrés dans les zones urbaines, et sont insuffisants pour satisfaire les besoins futurs et dépendent des professionnels itinérants.

## Introduction

The African region has 3% of the world's health workforce to tackle 24% of the global burden of disease.^1^ With only 11% of the world's population, the region accounts for half the world's maternal and child deaths and more than 90% of the world's deaths from malaria and HIV.^2^ The United Nation's Millennium Development Goals (MDGs) call for reducing under-five mortality by two-thirds and maternal mortality by three-quarters by 2015. Sub-Saharan Africa is unlikely to achieve those goals without significant increases in skilled human resources for health.^3, 4^ There is ample evidence that an increase in the number and quality of healthcare workers is associated positively with improved health outcomes.^1^ The shortage of human resources for health in sub-Saharan Africa is attributed to a number of complex factors including inadequate numbers trained, inappropriate skill mixes, inequitable distribution and migration.^1^ Migration to North America and Western Europe was the major cause of faculty loss in the Sub-Saharan African Medical Schools Survey.^5^ The exodus of skilled professionals in the midst of so much unmet health need places Africa at the epicentre of the global health workforce crisis, which contributes to weak health systems.^6^

### Botswana and human resources for health

Botswana is a landlocked country in Southern Africa with a landmass of 582 000 km^2^ and a population of just over 2 million.^7^ It has an annual government and total expenditure on health per capita of $246 and $382, respectively.^3^ There are 28 health districts made up of five urban, four rural and 19 rural districts, with one or more urban villages. For the purposes of this paper, urban areas are defined as ‘all settlements with a population of 5000 or more persons with at least 75% of labour force in non-agricultural occupations (subsistence farming)^7^ and villages are defined as settlements on tribal land, which have tribal administration and some basic services, including a primary care clinic, primary school and a post office. Combining these definitions, 27 villages were designated as ‘urban villages’ and this then leads to a distinction between districts that are completely rural and districts that are rural with one or more urban villages.^7^

Botswana's health services are mostly public with a small but growing private sector. The healthcare system is based on a primary healthcare model and services are provided through a network of three referral hospitals, seven district hospitals, 14 primary hospitals, three private hospitals, three mine hospitals, two mission hospitals, 265 primary care clinics (101 with maternity beds), a number of specialists and general private outpatient clinics (mostly in towns and some urban villages), 343 health posts and 861 mobile clinic sites. This has brought 95% of the total population (89% of the rural population) within eight kilometres of a healthcare facility. Access to a healthcare facility does not always translate to good health service, as many of the facilities are severely short staffed (MoH, Integrated Health Services Plan [IHSP] 2012, personal communication, Jan 23). Life expectancy at birth is estimated at 61 years.^3^ The infant and under-five mortality rates are 36 and 48 per 1000 live births respectively and the maternal mortality ratio (MMR) is 160/100 000 births.^3, 8^

The major employer of health workers in Botswana is the government. Prior to 2010, primary healthcare was managed by the Ministry of Local Government, but from April 01 2010, all healthcare was transferred to the Ministry of Health. A shortage of skilled and qualified healthcare workers remains one of the major bottlenecks toward the availability of accessible high quality healthcare in Botswana with 3.4 doctors and 28.4 nurses per 10 000 people.^3^ Training of health workers is achieved through a combination of in-country and foreign training institutions. In-country training is done at the eight Institutes of Health Sciences for diploma courses in nursing, midwifery, health education, laboratory, radiography and dental technology. The University of Botswana provides Bachelor's degrees and Master's degree courses in nursing, including specialist nursing, laboratory technology, environmental health and it will graduate its first class of doctors in October 2014. The Institutes of Health Sciences and the University of Botswana are public institutions. There is one private institution which offers training in phlebotomy, plaster, theatre and dental assistance, as well as training for health care auxiliaries.

Botswana has a paucity of readily-accessible, integrated and comprehensive information on human resources for health. A decision was therefore made to collate and analyse the available data from different sources, which are otherwise fragmented and sometimes difficult to access within the Ministry of Health (MoH), Ministry of Local Government and Ministry of Education and Skills Development. Professional regulatory bodies and multiple consultancy reports are also useful sources of information. The Department of Policy, Planning, Monitoring and Evaluation (DPPME) is responsible for human resources planning for the MoH. The Botswana Health Professions Council (BHPC) is the regulatory body for all doctors and allied health professionals, whilst the Nursing and Midwifery Council of Botswana (NMCB) is responsible for the registration and regulation of the practice of nurses and midwives.

The Ministry of Education and Skills Development (MESD) provides scholarships for tertiary education, including health sciences, both inside and outside the country. The Department of Tertiary Education Funding (DTEF) is responsible for administering these scholarships.

For the purposes of this study the following definitions of health cadres are used: ‘medical officers’ are generalist doctors without specialist training and ‘nurses’ refers to all nursing cadres including midwives, family nurse practitioners, community health nurses and all other nurses; ‘clinical support staff’ comprises health workers with minimal to no formal training that assist with patient care including theatre technicians, plaster technicians and nurse auxiliaries. Other health workers’ on the other hand refers to healthcare workers with training of up to 18 months, such as lay counsellors, phlebotomists, healthcare auxiliaries and health education assistants. ‘Pharmacists’ refers to pharmacists and pharmacy technicians (trained at diploma level). ‘Medical laboratory scientists’ refers to scientists (degree holders) and technicians (diploma holders).

### Aim and objectives

The aim of this study was to analyse the existing databases and report on the health workforce in Botswana in order to quantify and describe the situation with regard to the human resources for health. The specific objectives were:

1. To quantify the different types of health workers in Botswana.
2. To determine the vacancy rates for the different health worker categories at primary and higher levels of healthcare.
3. To compare the Botswana Human Resources Strategic Plan for Health (BHRSPH)'s recommended numbers of health workers to baseline levels at primary and higher levels of care.
4. To describe the density and distribution of the different health workers.
5. To describe the country of origin of health workers in Botswana.
6. To determine the number and place of training of Botswana health workers.

## Research methods and design

A compilation and analysis of the existing databases on human resources for health was carried out. This entailed face-to-face meetings with the DPPME of the MoH, the DTEF of the MESD, the BHPC, the NMCB and the in-country World Health Organization (WHO) office to explain the purpose of the study. Formal requests for their particular databases were subsequently made to each of the bodies. The data were collected from November 2011 to May 2012 and covered the period from 2007 to 2012.

The DPPME provided raw data on the 2009 head count of all their health workers as well as the 2012 database. The latter database also provided information on the distribution of the health workers in the country and the information on the number of these workers who were Batswana (citizens of Botswana). They also provided a number of consultancy reports, including the National Health Services Situational Analysis (NHSSA) as well as a number of policy and strategy documents including the National Health Policy (NHP), the BHRSPH (2008–2016) and the 10-year IHSP (2010–2020) and the Essential Health Services Package (EHSP). These are sources for some of the information in this study.

The BHPC provided data on the numbers, country of origin and distribution of the healthcare professionals registered with the council. The NMCB provided summary statistics of the nurses and midwives registered with the council, including the total numbers and the countries of origin.

The database on Batswana students, who were funded to study health science courses from 1997 to 2010, was sourced from the DTEF.

The in-country WHO office provided the Botswana Human Resources for Health Country Profile, which is the source of some of the information in this article.

Once the data were collected, attempts were made to improve their quality by seeking clarification on the definition of categories of health workers used by different sources. The databases were created over different time periods, with data collected for different purposes. They therefore had different types of information and none had enough information to answer all the aims of this study. Therefore, each database was used to answer different aspects of the study objectives, based on the relevance and perceived completeness of their information. The data were used to compile the different categories of health workers and calculate summary statistics. There was not enough information to calculate the change in the numbers or types or distribution of health workers over time.

## Results

### The quantities of health workers

In 2007 and 2008 MoH, Ministry of Local Government had a total staff complement of 6353, which included 2321 health workers (Table 1). The other employees were non-clinical non-technical support staff, administrators and non-clinical managers. The health workers included Bachelor's degree holders such as pharmacists, dentists, laboratory scientists, as well as technicians trained to diploma levels, including dental therapists, pharmacy and laboratory technicians (Tables 1 and 2). Medical Specialists at Primary Health Care level in 2007 and 2008 were Public Health Specialists. The recommended posts (Tables 1 and 2) are the estimated number of healthcare workers that will be required to provide health services in 2016 according to the BHRSPH. The projected 2016 vacancy rate is based on the number of filled positions in 2007 and 2008 (baseline) and the planned 2016 target for posts, assuming that there will be the same number of health workers in 2016 as at baseline.

**TABLE 1:** Primary care staff establishment 2007 and 2008 and recommendations for 2016.

Health worker category	Total posts	Filled posts	Current vacancy rate (% total posts)	Recommended posts	Projected 2016 vacancy rate (%) (FP/RP)
Dental therapists	2	2	0	0	-
Dieticians	0	0	0	26	100
Environmental health officers	75	70	7	164	57
Medical laboratory scientists	40	40	0	26	-
Medical officers	69	63	9	79	20
Nurses	1897	1816	4	3089	41
Other allied health workers	23	22	4	26	15
Pharmacists	89	78	12	392	80
Physiotherapists	2	2	0	0	-
Radiographers	4	6	0	0	-
Medical specialists	18	13	28	26	50
Social workers	19	19	0	26	27
Clinical support staff	38	38	0	0	-
Other health workers	45	40	11	868	95

**Totals**	**2321**	**2209**	**5**	**4722**	**53**

*Source*: Ministry of Health, Botswana Human Resources Strategic Plan for Health 2012, personal communication, January 23

**TABLE 2:** Secondary and tertiary hospital staff establishment 2007 and 2008 and recommendations for 2016.

Post category	Total posts	Filled posts	Current vacancy rate (% total posts)	Recommended posts	Projected 2016 vacancy rate (%) (FP/RP)
Dentists	28	21	25	40	48
Dieticians	17	15	12	17	12
Dental therapists	61	79	0	105	25
Environmental health officers	51	0	100	33	100
Medical laboratory technicians	248	254	0	587	57
Medical officers	244	195	20	356	45
Nurses	3552	2898	18	3147	8
Other allied health workers	189	134	29	267	50
Occupational therapists	10	11	0	28	61
Pharmacists	241	245	0	509	52
Physiotherapists	16	16	0	58	72
Psychologists	5	4	20	13	69
Radiographers	48	40	17	104	62
Medical specialists	137	89	35	192	54
Speech therapists	1	0	100	13	100
Social workers	2	38	0	65	42
Clinical support staff	89	233	0	387	40
Other health workers	13	18	0	1671	99

**Totals**	**4952**	**4290**	**13**	**7592**	**43**

*Source*: Ministry of Health, Botswana Human Resources Strategic Plan for Health 2012, personal communication, January 23 FP, Filled posts; RP, Recommended posts.

Prior to 2010, the MoH had a total workforce of 9652 which included 4952 health workers who were employed on the clinical platform in secondary and tertiary health institutions (Table 2). The MoH also employed 220 other health professionals who have been excluded from the analysis as they do not work on the clinical platform, but are teachers at the Institutes of Health Sciences (MoH, BHRSPH 2012, personal communication, Jan. 23)

### Vacancies

In 2007 and 2008 there were vacancies in seven of the 13 health worker categories in primary care. The vacancies were highest for medical specialists, pharmacists, medical officers and other health workers respectively (Table 1). The number of radiographers employed exceeded the number of established positions. The mean vacancy rate in primary care was 5%. There were very high vacancy rates in 10 of the 18 health worker categories in secondary and tertiary health care, the worst of which was for environmental health officers and speech therapists (Table 2). Dental therapists, social workers, clinical support staff and other health workers (Table 2) exceeded the number of established positions. The mean vacancy rate for secondary and tertiary healthcare services was 13%.

**FIGURE 1:**
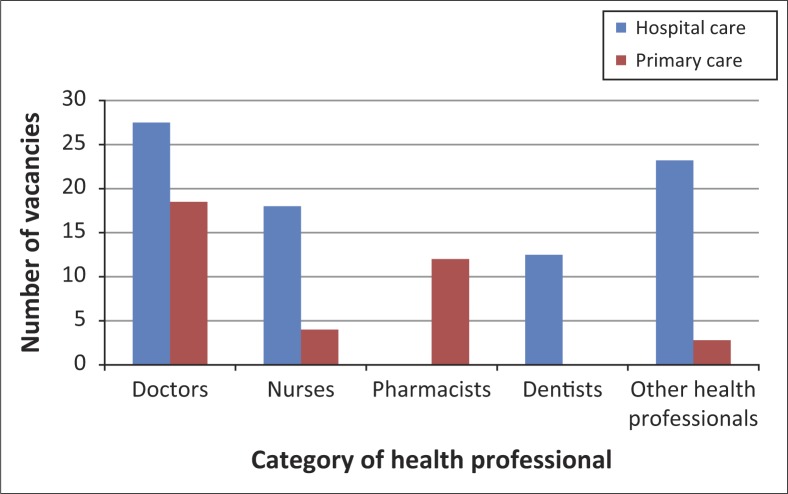
Comparison of vacancy rates in primary care versus secondary and/or tertiary hospital care 2007 and 2008. *Source*: Ministry of Health, Botswana Human Resources Strategic Plan for Health 2012, personal communication, January 23

**FIGURE 2:**
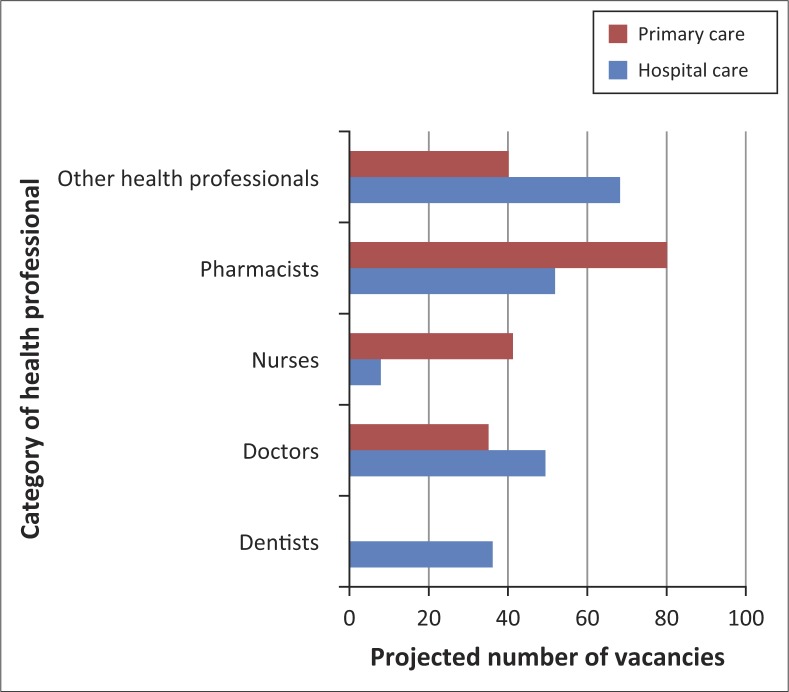
Comparison of projected 2016 vacancy rates for primary care versus secondary and/or tertiary hospital care. *Source*: Ministry of Health, Botswana Human Resources Strategic Plan for Health 2012, personal communication, January 23

The vacancy rates were much higher in secondary and tertiary healthcare services compared to primary health care (Figure 1), except for pharmacists where there were no vacancies in secondary/tertiary care compared to a 12% vacancy rate in primary care. Other health professionals refer to all other health workers excluding nurses, doctors, dentists and/or dental therapists, and pharmacists and/or pharmacy technicians (Figures 1 and 2).

### Recommended number of health workers for 2016 compared to baseline

To implement the BHRSPH, moderate increases in the numbers of healthcare workers will be required from baseline to the levels planned for 2016 (Tables 1 and 2, Figure 2). The highest increases at all levels of care will be in the ‘other health worker’ category with a projected 2016 vacancy rate of 95% in primary care and 99% in higher levels of care. Dieticians will be introduced in primary care and pharmacists increased significantly (Table 1). Some types of health workers are to be removed completely from primary care, including dental therapists, radiographers, physiotherapists and clinical support staff (Table 1). The number of nurses in secondary and/or tertiary care is to be reduced whilst those in primary care are to increase significantly (Figure 2). The mean 2016 vacancy rate, based on the 2007 and/or 2008 filled positions and the planned 2016 targets, is 53% in primary care compared to 43% in the secondary or tertiary care levels.

### Density and distribution of health workers

The densities of health workers were highest in urban areas and lowest in the rural districts with one or more urban villages (Table 3). In 2012, Gaborone, with 11% of Botswana's population, was home to 250 (34%) doctors and 1113 (17%) nurses whilst Francistown, with 5% of the population, had 100 (13%) doctors and 530 (8%) nurses (MoH 2012, personal communication, Jan. 23). In 2009, the proportion of doctors working in Gaborone and Francistown was 43% and 15% and for nurses, 11% and 4%, respectively (MoH, IHSP 2012, personal communication, Jan. 23).

**TABLE 3:** Density of health workers (per 10 000 population) in Botswana per district type.

District type	Population	Doctor	Nurses and/or midwives	Pharmacists	Dentists	Healthcare workers
Rural	98 816	3	26	1	0	45
Rural with urban villages	1 515 181	2	22	1	1	35
Urban	424 231	9	77	4	1	115

**Total**	**2038228**	**4**	**42**	**2**	**1**	**65**

*Source*: Ministry of Health, Department of Policy, Planning, Monitoring and Evaluation 2012, personal communication, April 04

### Country of origin of Botswana health workers

In 2012, 13 713 health workers were registered with the BHPC and NMCB. The BHPC had 4416 registrants whilst the NMCB had 9297 (Table 4). There were 1820 doctors registered with the Botswana Health Professions Council. Batswana (native-born and naturalised citizens of Botswana) made up 41% of all health professionals registered with BHPC and 84% of the nurses registered with NMCB (Table 4). Only 21% of the doctors registered with the BHPC were Batswana compared with 84% of nurses (Table 4). Batswana were also in the minority amongst physiotherapists (39%), radiographers (13%) and optometrists (35%) (BHPC 2012, personal communication, May 16).

**TABLE 4:** Health workers who are citizens of Botswana as compared to the total number of health workers in Botswana.

Health workers	Total number	Batswana	

		*n*	%
Doctors	1820	382	21
Nurses	9297	7845	84
Dentists and/or dental therapists	296	223	75
Pharmacists and/or pharmacy technicians	764	402	53
Other health workers	3645	2226	61

**Total**	**13 713**	**9635**	**70**

*Source*: Ministry of Health, Botswana Health Professions Council and Nursing and Midwifery Council of Botswana 2012, personal communication, May 16

The major contributors to health professionals registered with the BHPC in 2012 were Botswana, Zimbabwe, Democratic Republic of Congo (DRC) and Zambia (Table 5). Zimbabwe mainly contributed doctors (*n* = 191), laboratory scientists (*n* = 127), physiotherapists (*n* = 34), radiographers (*n* = 90), pharmacy technicians (*n* = 49), pharmacists (*n* = 52) and laboratory technicians (*n* = 43), whilst Zambia mainly contributed doctors (*n* = 73), laboratory technicians (*n* = 28), pharmacy technicians (*n* = 42), physiotherapists (*n* = 19) and radiographers (*n* = 77). The DRC was a significant source of doctors (*n* = 333) (BHPC 2012, personal communication, May 16).

**TABLE 5:** Country of origin of health professionals registered with the Botswana Health Professions Council in 2012.

Country of origin	All healthcare workers (*N* = 4416)		Doctors (*N* = 1820)	

	*n*	%	*n*	%
Botswana	1790	40	382	21
Zimbabwe	630	14	191	10
Democratic Republic of Congo	351	8	333	18
Zambia	266	6	73	4
India	199	4	97	5
Nigeria	174	4	96	5
Republic of South Africa	140	3	101	6
United States of America	110	2.5	70	4
Kenya	109	2.5	37	2
Tanzania	101	2.3	65	4
Ethiopia	72	1.6	69	4
China	59	1.3	55	3
Uganda	56	1.3	40	2
Other African	90	2.0	48	3
Other non-African	269	6.1	163	9

*Source*: Ministry of Health. Botswana Health Professions Council 2012, personal communication, May 16

### Training of health workers

Between 1997 and 2010, an estimated 7154 Batswana graduated as health professionals with government financial support (Table 6). Of these, 1665 (23%) graduated from universities in 21 countries in Europe, North America, South America, Australia, Africa and South East Asia. This latter group included doctors (48%), pharmacists (10%), dentists (11%) and nurses (11%), as well as physiotherapists, nutritionists, dieticians, biomedical engineers, optometrists, occupational therapists, radiographers, audiologists and speech therapists and occupational health and safety officers.

**TABLE 6:** Numbers and place of training of state-supported health professionals graduated between 1997 and 2010.

Health care trained	*N*	Botswana		Other countries	
	
		*n*	%	*n*	%
Dentists^1^	272	89	33	183	67
Pharmacists^1^	350	190	54	160	46
Nurses^1^	5031	4846	96	185	4
Doctors	802	0	0	802	100
Other health workers	699	364	52	335	48

**Total**	**7154**	**5489**	**77**	**1665**	**23**

*Source*: Department of Tertiary Education Funding 2012, personal communication, April 06

1 Bachelors and diploma levels.

## Discussion

The density of doctors and nurses per 10 000 population was 4.3 and 41.3, respectively, using the MoH database of 2012. The densities of doctors and nurses in rural districts were significantly lower than urban districts. This is in keeping with the findings in many low- and middle-income countries.^9, 10, 11^ Disparity in the distribution with significant skewing favouring urban areas is also well described in many settings.^12, 13^ The densities are a little higher in purely rural districts because these are very sparsely populated, with some clinics attending to as few as four patients a day (MoH, IHSP 2012, personal communication, Jan 23). The arrangement is necessary to meet the government targets of bringing 95% of the population within eight kilometres of a health facility.^8^ The density of doctors and nurses in Botswana was similar to Namibia and higher than many countries in the region such as Zambia and Zimbabwe, but lower than South Africa.^4^ A number of African countries are said to have a critical shortage of health workers, with fewer than 2.3 health workers per 10 000 population.^1^ Although Botswana is not amongst the countries with a critical shortage of health workers, it still has far fewer numbers than countries with established health systems such as the United Kingdom (UK).^1^

There is evidence that adequate numbers of health workers are associated with positive health outcomes.^1^ Botswana's life expectancy at birth as well as maternal and under-five mortalities were similar to Namibia, which had equivalent densities of health workers, but much better than Zimbabwe, Zambia, Uganda, Mali and Sudan, all of which had much lower health worker densities.^4^ Surprisingly, Botswana's health indices were better than those of South Africa although the latter had higher health worker densities.^4^ Botswana's health indices were much worse than the UK, which was in keeping with the UK's higher densities of health workers.^4^ There are, of course, other factors that also contribute to these differences.

The mean vacancy rate in primary healthcare was less than that for secondary and tertiary care. This is different from many low-income countries, which have very high vacancy rates in primary healthcare compared with higher levels of care.^14, 15^ However, with far fewer posts now than projected for 2016 in primary care compared with secondary and/or tertiary care, the number of health workers required in primary care was grossly underestimated. This shows that the system has been skewed toward recruitment in secondary and/or tertiary care settings since only 34% of filled posts and 32% of total posts were in primary care settings. This is the inverse of what would be needed in a more efficient system based on universal access to good quality primary healthcare.^15^

Since about 2006 and 2007, the government has been rolling out the management of patients with HIV infection to primary care, which may explain the significant projected increases in the numbers of pharmacists, nurses, other health workers and medical specialist, as well as the introduction of dieticians. It is not clear from the data as to which medical specialties were intended for primary care, but according to a more recent strategy document, the EHSP (2010–2020), Family Physicians are to be based in Primary Hospitals and not in primary care clinics (MoH, IHSP 2012, personal communication, Jan. 23). Primary Hospitals are part of secondary-level care with less than 100 in-patient beds and usually do not have specialist doctors. The planned increases in other healthcare workers reflects the intention to shift a number of tasks currently done by nurses and doctors to health workers with shorter training such as health education assistants and lay counsellors as well as an increase in the links between primary care and the community-based services (MoH, EHSP 2012, personal communication, Apr. 06).

Task shifting, when properly implemented and with adequate supervision, can improve significantly the ability of countries to increase their health workforce, improve skill mixes and reduce international migration. The challenges are maintaining the quality of care and patient safety.^16^ Removing radiographers, physiotherapists and dental therapists from primary care is likely to have an adverse effect on the ability of the health system to provide equitable and accessible care. The increases in the number of health workers at all levels of the health services mandates significant increases in the training of nurses, other health workers, pharmacists and doctors, including medical specialists. With only 21% of the doctors in the country being Batswana (citizens of Botswana), Botswana is heavily dependent on migrant health workers, mostly from countries such as the DRC, Zimbabwe and Zambia, which are plagued with significant political and/or economic hardships. This overdependence on an expatriate health workforce puts the country at significant risk as these health workers are likely to want to return to their countries as situations improve, or to migrate to higher-income countries.

Local training appears to reduce significantly the dependence on expatriate health workers. The dependence on foreign training institutions for doctors has proven to be ineffective and unsustainable as the majority of the graduates have tended to remain in the host countries on completion of training, which tend to be higher-income countries with better salaries and more career development opportunities (DTEF 2012, personal communication, Apr. 06). Some of the host countries also see the foreign students as a potential solution to their own human resources for health shortages. Australia, for instance is exploring the potential contribution of international medical students in Australian medical schools to the Australian medical workforce.^16^ The establishment of a local medical school is intended to reduce the dependence on expatriate doctors. This will need to be monitored closely, however, as many developing countries have continued to experience a reduction in the numbers of doctors despite in-country training, because of migration to higher-income countries.^17^

### Limitations

The major limitation of the study was the incompleteness and inaccuracy of the data, which made it difficult to get a reliable estimate of the quantities, densities and distribution of health workers. An unidentifiable number of health professionals on the BHPC register work outside the country. None of the databases had detailed information on health workers in other sectors of the health system such as the private sector and non-governmental organisations. Therefore, an accurate estimation of the total health worker density is not possible. Although the data span from 2007 to 2012, they are not adequate to calculate trends in human resources for health.

None of the databases available had information on the distribution of health workers according to the level of care and the data were not disaggregated to the level of the individual clinic or hospital. The only data on vacancy rates are very old, being from 2007 and 2008.

### Recommendations

Botswana has an urgent need to establish a comprehensive human resource information system that will be resourced adequately in order to keep it up-to-date in real-time and accessible to all stakeholders. The information should differentiate between the different levels of healthcare and should also be disaggregated to the level of the healthcare facility. The disparity in the distribution of health workers between the rural and urban districts should be addressed as a matter of priority in order to achieve universal access to healthcare. For an optimally-functioning health system, all levels of care must be resourced both adequately and appropriately. Research is needed so as to find out how health workers can be attracted to and retained in Botswana's rural areas in order to address the disparity.

Training of doctors, including medical specialists, nurses, pharmacists and pharmacy technicians, as well as lay counsellors and health educations assistants, needs to be increased in order to meet the planned EHSP and BHRSPH requirements. All training should be supported by strategies for attraction and retention so as to reduce the heavy dependence of the country on an expatriate health workforce, especially doctors.

## Conclusion

The MoH is the sole employer for the public health service. In 2007 and 2008 there were relatively high vacancy rates for different types of health workers, especially in secondary and/or tertiary care. Plans to increase significantly the numbers of health workers at all levels of health care by 2016, if not coupled with a significant injection of new health workers, will exacerbate the vacancy rates. The density of health workers in Botswana is relatively high compared to its neighbours, but this is still too low to enable the country to achieve its MDGs. Similar to many countries, health workers tend to be more densely distributed in urban compared with rural areas. Botswana has a worrisomely high dependence on an expatriate health workforce and, until recently, out-of-country training of doctors and dentists and allied health professionals. Attraction and retention of internationally-trained health workers has been unsuccessful and localisation of training is intended to more effectively meet the country's needs. In order to meet the country's human resources for health needs in a sustainable manner, more health workers will need to be trained, recruited and retained.
